# Sebaceous neoplasms: Just the thin end of the wedge

**DOI:** 10.1002/ccr3.2627

**Published:** 2020-01-22

**Authors:** Aikaterini Kyriakou, Nikiforos Galanis, Eliza Stavride, Aikaterini Patsatsi, Elizabeth Lazaridou, Eleftherios Tsiridis

**Affiliations:** ^1^ 2nd Department of Dermatology and Venereology “Papageorgiou” General Hospital Aristotle University School of Medicine Thessaloniki Greece; ^2^ Department of Orthopaedics “Papageorgiou” General Hospital Aristotle University School of Medicine Thessaloniki Greece; ^3^ Department of Radiology Papageorgiou General Hospital Thessaloniki Greece

**Keywords:** malignancy, Muir‐Torre syndrome, sebaceous tumors

## Abstract

The presence of sebaceous neoplasm should alert physicians to thoroughly investigate for underlying malignancies. Awareness on MTS should be raised within physicians, since this may be just the thin end of the wedge.

## Graphical abstract

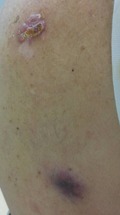

The presence of sebaceous neoplasm should alert physicians to thoroughly investigate for underlying malignancies. Prompt diagnosis of MTS (Muir‐Torre syndrome) and annual surveillance for internal malignancy is crucial for patients and family members. Awareness on MTS should be raised within physicians, since this may be just the thin end of the wedge.

A 66‐year‐old male presented to the emergency room with severe sternoclavicular pain exacerbated by movement. Upon clinical examination, multiple, deep‐seated, and gradually enlarging nodules on patient's back were found (Figure 1). In the course of time, some of the lesions had become ulcerated and crusted. Moreover, a sizable keratoacanthoma located on the right arm was recognized (Figure 2). No significant personal medical history was reported, apart from hypertension, while his family history of internal malignancies was uncertain. Skin lesions were biopsied, and the histopathological examination of the back lesions was consistent with sebaceous carcinoma, while the arm lesion revealed a keratoacanthoma with sebaceous differentiation. Upon these findings, the diagnosis of Muir‐Torre syndrome (MTS) was considered. CT scan was conducted and revealed lung cancer with multiple bone metastases (Figure 3A,B). MTS is a rare genodermatosis defined by the presence of sebaceous neoplasms and visceral malignancies.^1^, ^2^ Even though rare, sebaceous tumors are the most indicative cutaneous sign of MTS.^1^ The most common visceral malignancy is colorectal adenocarcinoma. Other reported cancers include those of the endometrium, ovary, small bowel, pancreas, hepatobiliary tract, brain, upper uroepithelial tract, breast, and lung. The presence of a sebaceous neoplasm should alert physicians to thoroughly investigate for underlying visceral malignancies.^1^ Prompt diagnosis of MTS and annual surveillance for internal malignancy is crucial for both patients and family members.^1^, ^2^ Knowledge and awareness on MTS should be raised within physicians, especially when examining patients with multiple cancerous lesions with no obvious explanation, since these may be just the thin end of the wedge.

**Figure 1:**
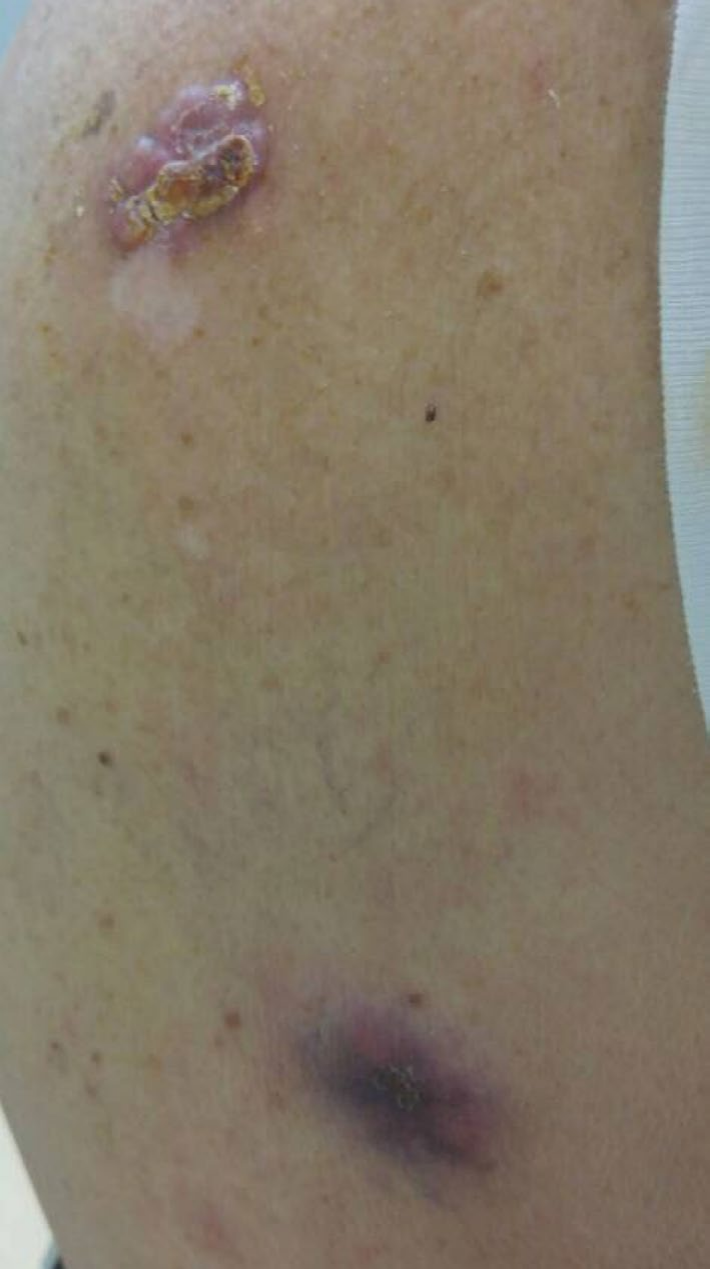
Two lesions compatible with sebaceous carcinomas located on the back

**Figure 2:**
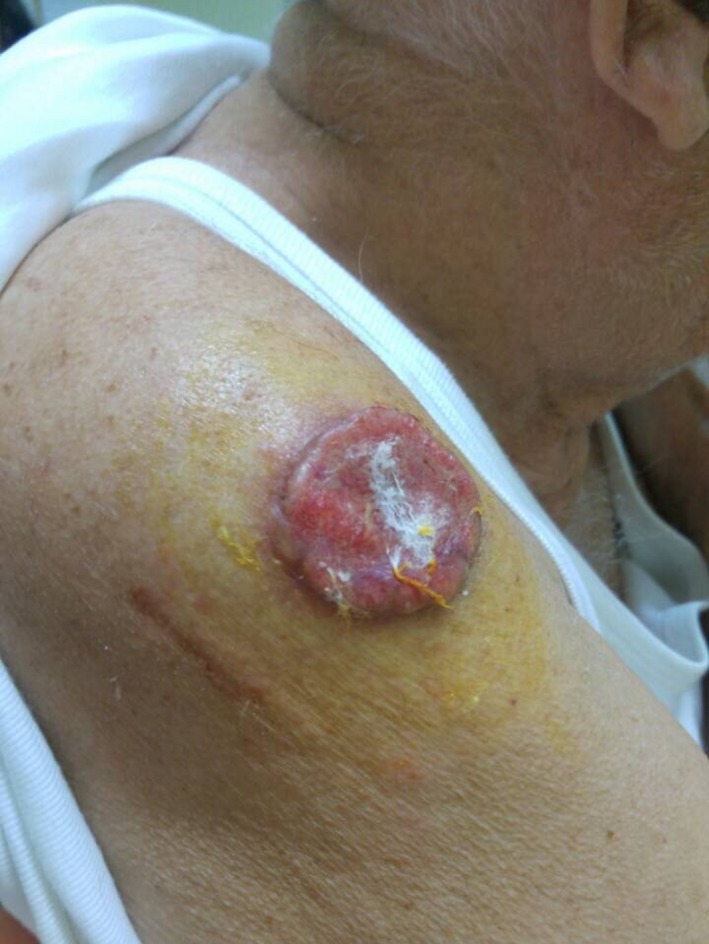
Keratoacanthoma with sebaceous differentiation located on the right arm

**Figure 3:**
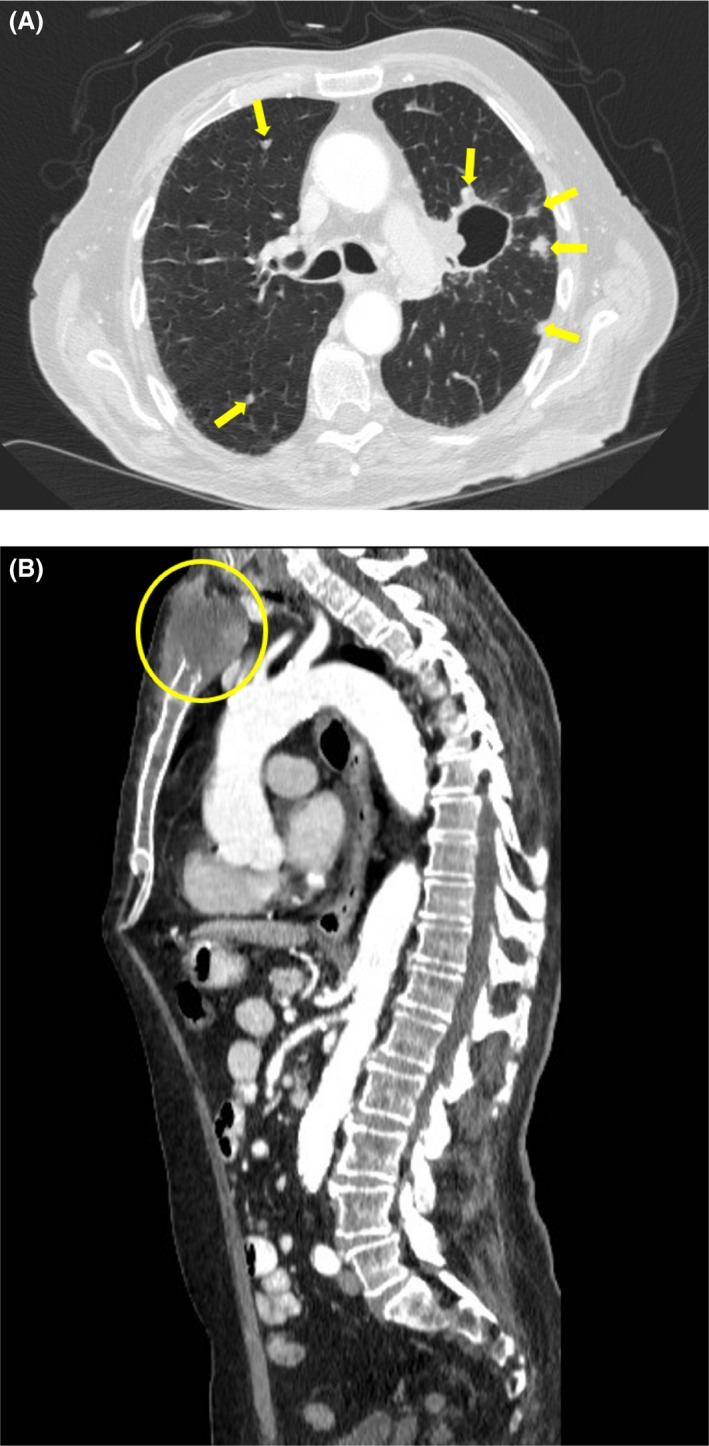
A, Axial computed tomography image showing multiple metastatic nodules in the lung parenchyma. B, Sagittal computed tomography image demonstrating a big metastatic lesion eroding the sternum

## CONFLICT OF INTEREST

None declared.

## AUTHOR CONTRIBUTION

AK, NG: Drafted the manuscript, obtained the photographs, and contributed to patient's care and diagnosis. ES, AP, EL, IT: Critically reviewed the paper and contributed to diagnosis.
